# The prevalence and associated factors of undernutrition among under-five children in South Sudan using the standardized monitoring and assessment of relief and transitions (SMART) methodology

**DOI:** 10.1186/s40795-021-00425-3

**Published:** 2021-05-28

**Authors:** Jackline Kiarie, Sarah Karanja, Julius Busiri, Diana Mukami, Colleta Kiilu

**Affiliations:** 1 Amref Health Africa in Kenya, Nairobi, Kenya; 2 Amref Health Africa Headquarters, Nairobi, Kenya; 3 Amref Health Africa in South Sudan, Juba, South Sudan

**Keywords:** Child Undernutrition, Wasting, Underweight, Stunting, Food insecurity, Conflict regions

## Abstract

**Background:**

Conflict regions bear the heaviest brunt of food insecurity and undernutrition. South Sudan is one of the fragile countries following years of conflict that led to large displacements. Moderate to severe undernutrition among under-five children has been associated with elevated morbidity and mortality. This study, therefore, was conducted to assess the magnitude and factors influencing undernutrition (wasting, underweight and stunting) among children aged 6 to 59 months in Yambio County, South Sudan.

**Methods:**

A cross-sectional study was conducted from 26 October to 6 November 2018 in Yambio County, South Sudan among 630 children aged 6–59 months from the 348 households surveyed in 39 clusters using two-stage cluster sampling design. Data were collected using questionnaires and nutritional anthropometric measurements. The Standardized Monitoring and Assessment of Relief and Transitions (SMART) Methodology was followed to obtain the prevalence of wasting, underweight and stunting based on respective z scores and according to the 2006 world health organization child growth standards. Data were exported to Stata version 16 for further analysis. Bivariate analysis of independent variables and undernutrition was done using binary logistic regression. Mixed effects logistic regression analysis was conducted to control for possible confounders and account for random effects at household and cluster levels. Unadjusted and adjusted odds ratios (cOR and aOR) with 95% confidence intervals (CI) and *p*-values were computed. *P*-values of ≤0.05 were considered statistically significant.

**Results:**

The prevalence of undernutrition explained by wasting (weight-for-height Z-score (WHZ) < − 2), underweight (weight-for-age z-scores (WAZ) < − 2) and stunting (height-for-age z-scores (WHZ) < − 2) were 2.3% (1.3–4.1, 95% CI), 4.8% (3.1–7.5, 95% CI) and 23.8% (19.1–29.2, 95% CI). Male sex (aOR [95% CI], *p*-value: 5.6 [1.10–30.04], *p* = 0.038), older child’s age (aOR [95% CI], *p*-value: 30.4 [2.65–347.60], *p* = 0.006) and non-residents (cOR [95% CI], *p*-value: 4.2 [1.4–12.2] *p* = 0.009) were associated with increased risk of wasting. Household size (cOR [95% CI], *p*-value: 1.09 [1.01–1.18] *p* = 0.029) and younger child age (cOR [95% CI], *p*-value: 4.2 [1.34–13.23] *p* = 0.014) were significantly associated with underweight. Younger child age (aOR [95% CI], *p*-value: 5.4 [1.82–16.44] *p* = 0.003) and agricultural livelihood (aOR [95% CI], p-value: 3.4 [1.61–7.02] *p* = 0.001) were associated with stunting.

**Conclusion:**

Based on a cut off of less than − 2 standard deviations for 2006 World Health Organization (WHO) child growth standards, the wasting prevalence was very low, underweight prevalence was low while stunting prevalence was high. The county lies in the only livelihood region in South Sudan with bimodal reliable rainfall pattern and it seems that the impact of the 2016 conflicts that lead to large displacements may not have greatly affected under-five undernutrition. Interventions targeted at improving food diversity, increasing nutrition knowledge and enhancing resilience in male children might reduce undernutrition. In the short-term, investment in continued surveillance of nutritional status should be a main focus.

## Background

The burden of undernutrition remains alarmingly high and impacts child growth and survival rate, especially in low- and middle-income countries [1, 2]. Globally 22.2% (150 million) of children are stunted and 7.5% (50.5 million) are wasted [1].. Global hunger has been on the increase after more than two decades of decline, with one of the worst-case scenarios reported in South Sudan where famine was declared in February 2017 [3]. The surge in global hunger was mainly due to conflict and this explains why conflict regions bear the heaviest brunt of food insecurity and malnutrition [3–5]. Over 489 million of the 815 million hungry people in 2016 lived in countries affected by conflict and these countries also accounted for 75% (122 million) of the world’s stunted under-5 year olds [3, 4]. Globally, around 2 billion people live in countries affected by fragility, conflict and violence, and South Sudan is one of the countries classified by World Bank as fragile [6].

Conflicts adversely impact food security through mass displacements, deep economic recessions, increased inflation, unemployment and eroded finances for social protection and health. In regions where agriculture is the main livelihood, conflicts affect food production, harvesting, processing, transportation and marketing resulting to poor resilience. Consequently, deteriorations in food security can exacerbate tensions and risks of conflict [3, 4]. In these settings, under-five children face increased risk of malnutrition and its related complications [7]. However, undernutrition among under-five children is also influenced by many other factors. Sociodemographic factors including mothers’ characteristics like age, education level, occupation, birth intervals and child characteristics like age and sex have been reported in some studies [8–12]. Low child birthweight, not up-to-date immunization, poor maternal nutritional status, food related cultural practices affecting food intake, early introduction of foods, pre-harvest season (compared to post-harvest), environmental factors like poor hygiene, diseases (chronic or being sick a few weeks before the survey), low dietary intake and poor access to food among others have also been associated with malnutrition among under-five children [8, 13–16].

Moderate to severe malnutrition has been associated with elevated mortality [17] and global estimates of nearly half of all deaths in under-five children has been attributed to undernutrition [1]. There is an epidemiologic synergism between malnutrition and morbidity [17, 18]. Survivors of undernutrition may suffer from impaired physical and intellectual development, may have higher levels of chronic illnesses later in life and malnourished females are more likely to give birth to low-weight babies [16, 19, 20]. It is therefore paramount to understand the extent and factors influencing under-five undernutrition to inform appropriate interventions in conflict regions. The objective of this study was to determine the prevalence and identify the sociodemographic, maternal, and child predictors of wasting, underweight and stunting among children aged 6 to 59 months in Yambio County, South Sudan.

## Methods

### Study design

This was a cross-sectional study using the Standardized Monitoring and Assessment of Relief and Transitions (SMART) methodology. The survey applied a two-stage cluster sampling design based on probability proportional to population size and obtained a population-representative sample. SMART is a standardized methodology of undertaking surveys which collect information on two vital public health indicators for assessing the severity of a humanitarian crisis: the nutritional status of children under-five and the mortality rate of the population [21].

### Study setting

The study was conducted in Yambio County in Gbudue State, South Sudan. Yambio County lies East of Gbudue State, bordering Nzara County to the west, Ibba County (Maridi State) to its east, Wulu County (Lakes State) to north and Democratic Republic of the Congo to the South. Yambio County consists of 32 Bomas in 5 *Payams* (administrative division), namely, Yambio town, Li-Rangu, Bangasu, Gangura and Nadiangere [22]. At the time of the survey, access was a challenge in Li-Rangu payam (with 4 bomas); Nadiangere payam (with 3 bomas) and 2 bomas in Bangasu payam (Ukuo & Nyaka) due to insecurity and this slowed down the survey in some villages.

Like many other areas in the greater Equatoria, Yambio was relatively stable following the outbreak of the December 2013 crisis. However, beginning early 2015, growing tensions between local communities, various armed militias and government security services led to increasing insecurity, conflict and multiple displacements. The known recent displacements took place in Western part of Yambio, Rimenze. Yambio was generally considered stable and hosting refugee communities from neighboring country of Democratic Republic of the Congo [23]. This study was conducted from 26 October to 6 November 2018.

### Study population

All children aged 6–59 months living in in Yambio County. Eligible children from nomadic areas with very sparse population and inaccessible households/villages due to long distance (like > 4 h one way) or insecurity were excluded from the survey.

### Sample size and sampling techniques

Emergency Nutrition Assessment (ENA) for SMART software (July 9th, 2015 version) was used to calculate the required minimum sample size. This was determined on the basis of estimated prevalence rates of malnutrition from neighbouring Maridi County SMART survey which was conducted in December 2016, a desired precision of 2.5% and design effect of 1.5, average household size of 6, percentage of under-fives in the population (21%) and 5% estimated non-response rate [24]. Based on these parameters, 502 households were visited within 39 clusters and 630 children aged 6–59 months were included in the survey. Of the 502 households surveyed, 348 had children aged 6–59 months.

### Data collection and variable measurement

This was part of a larger nutrition and mortality survey using SMART survey data collection tools in Yambio county. A 5-day data collection training including anthropometric standardization test was conducted. Data on the nutritional status of children under-five was used for this study.

Sociodemographic information and anthropometric measurements were collected from all children 6–59 months old in the sampled households. Age was obtained from the child’s immunization card, birth certificate or birth notification. A local calendar of events was used to estimate the age in case of missing documents. Children were weighed using digital weighing scales with minimal or no clothing and recorded to the nearest 0.1 kg. A height board was used to measure the height of children above 2 years of age and the length of children below 2 years of age. While ensuring minimal or no movement of the child and maintaining height readings at eye level, height was recorded to the nearest 0.1 cm. Bilateral oedema was assessed by the application of moderate thumb pressure for at least 3 s on both feet. If a depression was formed upon pressure application, then presence of bilateral oedema was confirmed.

Measles immunization was assessed by checking for measles vaccination on Expanded programme of immunization cards or by recall for children aged 9–59 months. Vitamin A supplementation was assessed by asking mothers/caretakers whether the child had received Vitamin A in the last 6 months. EPI cards and Vitamin A capsule was shown to caregivers of children aged 6–59 months to aid in recall. Two-weeks retrospective morbidity data was collected from the mothers/caretakers. Information on usage of long-lasting insecticide treated nets on the night preceding the survey and children (12–59 months) who were dewormed in the last 3 months was collected. Other variables including child’s sex, household size, household head, main livelihood activity and residence status were captured.

### Data analysis

Descriptive analysis of the study population was presented using means (standard deviations) and frequencies (proportions). Anthropometry data was analyzed using ENA for SMART to obtain the prevalence of acute malnutrition (wasting), underweight and stunting based on respective z scores. Anthropometric measurements were converted into weight-for-age z-score (WAZ), height-for-age z-score (HAZ), and weight-for-height z-score (WHZ) according to 2006 World Health Organization (WHO) child growth standards [25]. A cut off of less than − 2 standard deviations was used to determine wasting, underweight and stunting. The following SMART flags were used in the final analysis to exclude z scores with extreme values from observed mean: WHZ − 3 to 3; HAZ − 3 to 3; WAZ − 3 to 3.

Thereafter, data was exported to Stata version 16 for further analysis [26]. Descriptive statistics were used to report the prevalence of undernutrition (wasting, underweight and stunting). Additionally, the findings were presented using narrative, tables and figures. Bivariate analysis of independent variables and undernutrition (wasting, underweight and stunting) was done using binary logistic regression. Mixed effects logistic regression modelling was carried out to determine the significant predictors of wasting, underweight and stunting and to control for possible confounding variables (like age and main livelihood among others) while accounting for random effects at household and cluster levels. Unadjusted and adjusted odds ratios (aOR) with 95% confidence intervals (CIs) and *p*-values were generated to determine the significant associated factors for undernutrition.

All data generated or analysed during this study are included in this manuscript and its supplementary information files.

## Results

### Sociodemographic characteristics of children and parents

A total of 630 children aged 6–59 months were assessed for their nutritional status using anthropometric measurement from the 348 households surveyed in 39 clusters, with 310 (49.2%) being girls. Sex distribution was equal in the survey with a sex ratio of 1.03 (*p* = 0.690). Twenty-four percent of the sampled children did not have an exact documented birthday.. Age and sex distribution were as shown in Table 1. There was a significant age ratio of 6–29 months to 30–59 months of 1.06 against an expected value of 0.85 (*p* = 0.006) suggesting that more children 6–29 months in the sample than anticipated. The average household size was 9.2 ± 4.4, with 505 (80.2%) of the under-five children coming from male headed households. Over 20 % of the children (20.5%) were not residents of Yambio County, with 111 being internally displaced persons, 15 being refugees, and 3 being returnees. The distribution of the main household livelihood activities of the 630 under-five children were 82.4% agricultural (livestock and farming), 8.4% traders, 5.2% skilled laborer’s and 4% casual laborers. The sociodemographic characteristics of the respondents were as shown in Table 1.

**Table 1:** Characteristics of respondents in Yambio County, South Sudan, in 2018

Variables	Frequency (Percent) or Mean ± Standard deviation
Gender	
Female	310 (49.2)
Male	320 (50.8)
Age (Months)	
6–23	237 (37.6)
24–59	393 (62.4)
Household head	
Male	505 (80.2)
Female	125 (19.8)
Household size (household-level indicator)	9.2 ± 4.4
Resident status (household-level indicator)	
Resident	501 (79.5)
Non-resident	129 (20.5)
Main livelihood activity in the last 3 months (household-level indicator)	
Agricultural	519 (82.4)
Non-agricultural	111 (17.6)
Children (6–59 months) with Vitamin A supplementation in the last 6 months	
No	181 (28.7)
Yes	449 (71.3)
Children (9–59 months) immunized against measles	
No	69 (11.5)
Yes	529 (88.5)
Children (6–59 months) with illness in the previous 2 weeks before the survey	
No	292 (47.5)
Yes	323 (52.5)
Children (6–59 months) using Long Lasting Insecticide Treated Nets during the night preceding the survey	
No	192 (30.9)
Yes	429 (69.1)
Children (12–59 months) who were dewormed in the last 3 months	
No	301 (54.2)
Yes	254 (45.8)

Note: household-level indicators were duplicated if there were multiple children in the same household

### Healthcare utilization

71.3% (449/630) of children aged 6–23 months had received vitamin A supplementation in the previous 6 months before the survey. Measles immunization based on immunization card and/or recall for children aged 9–59 months was 88.5% (529/598) while deworming for children aged 12–59 months in the last 6 months preceding the survey was 45.3% (254/561). Nearly 7 out of 10 children (429/621) were reported to have slept under a mosquito during the night preceding the survey. Half of the children (51.3%, 323/615) had suffered from at least one illness in the previous 2 weeks before the survey, with majority (45.8%, 146) having fever, a third (29.1%, 94) reporting diarrhoea and a few reporting cough (23.2%, 75). The healthcare utilization characteristics of the respondents were as shown in Table 1.

### Prevalence of undernutrition

**Fig. 1:**
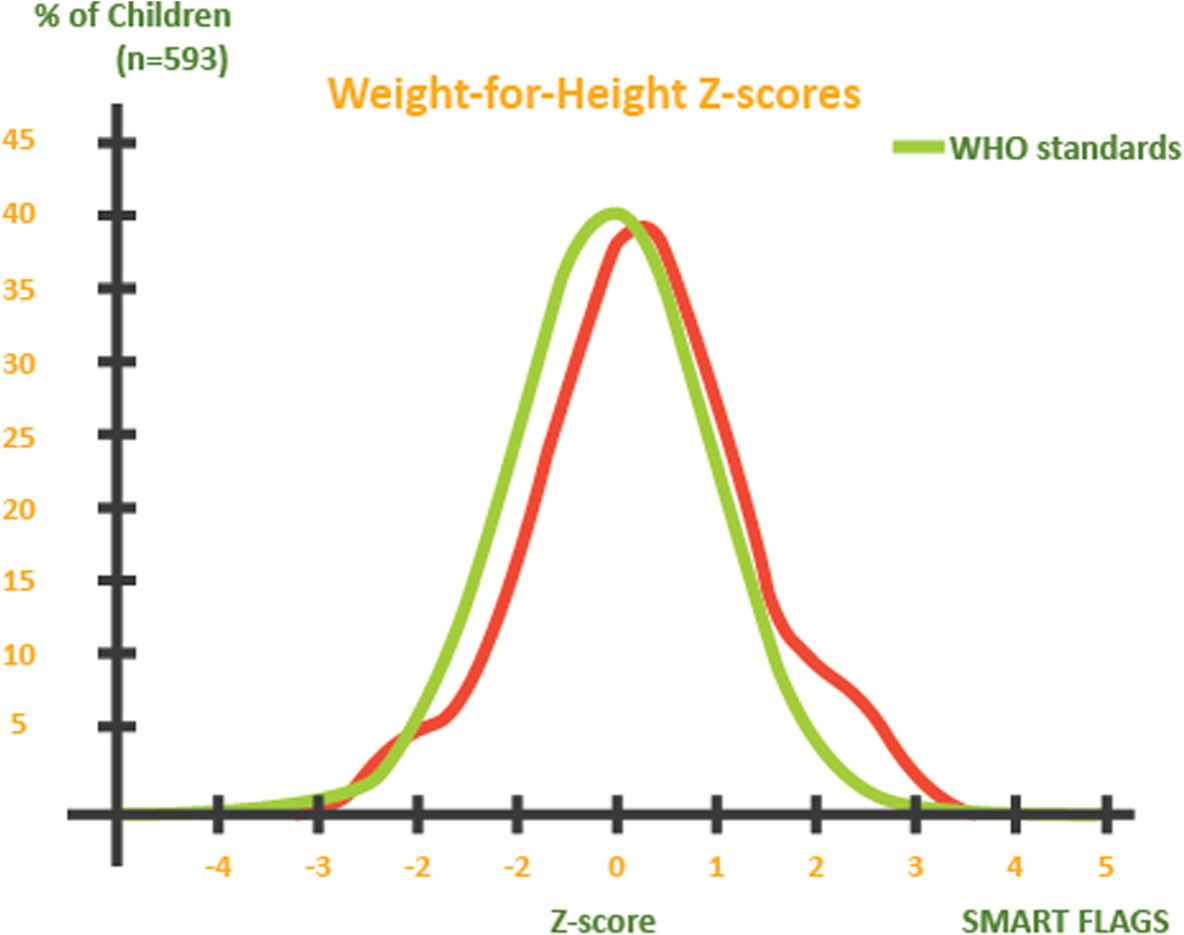
Distribution of weight-for-height z-scores compared to 2006 WHO standards among children below 5 years of age in Yambio County, South Sudan, in 2018 (*n* = 615)

A total of 14 under-five children had wasting which translated to an overall global acute malnutrition (wasting) using WHZ of 2.3% (1.3–4.1, 95% CI). There were no cases of severe acute malnutrition. The prevalence of edema was 0.0%. The distribution of weight-for-height z-scores compared to 2006 WHO standards among children below 5 years of age was as shown in Fig. 1.

**Fig. 2:**
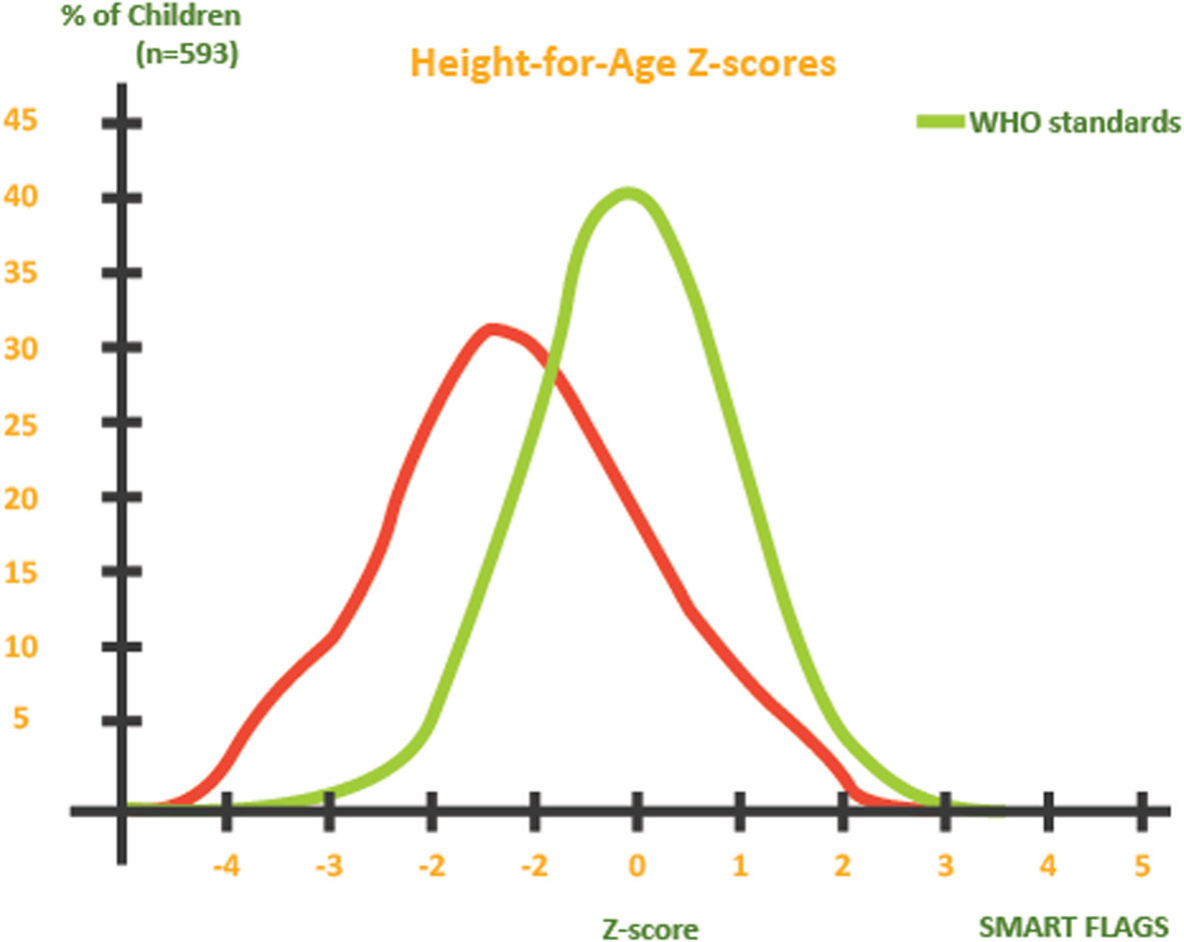
Distribution of height-for-age z-scores compared to 2006 WHO standards among children below 5 years of age in Yambio County, South Sudan, in 2018 (*n* = 593)

The overall prevalence of underweight based on weight-for-age z-scores (<− 2 z-score) in Yambio County was 4.8% (3.1–7.5, 95% CI), moderate underweight (<− 2 z-score and > = − 3 z-score) of 3.7% (2.4–5.7, 95% CI) and severe underweight (<− 3 z-score) of 1.1% (0.5–2.4, 95% CI). Figure 2 shows the distribution of weight-for-age z-scores compared to 2006 WHO standards among children below 5 years of age.

**Fig. 3:**
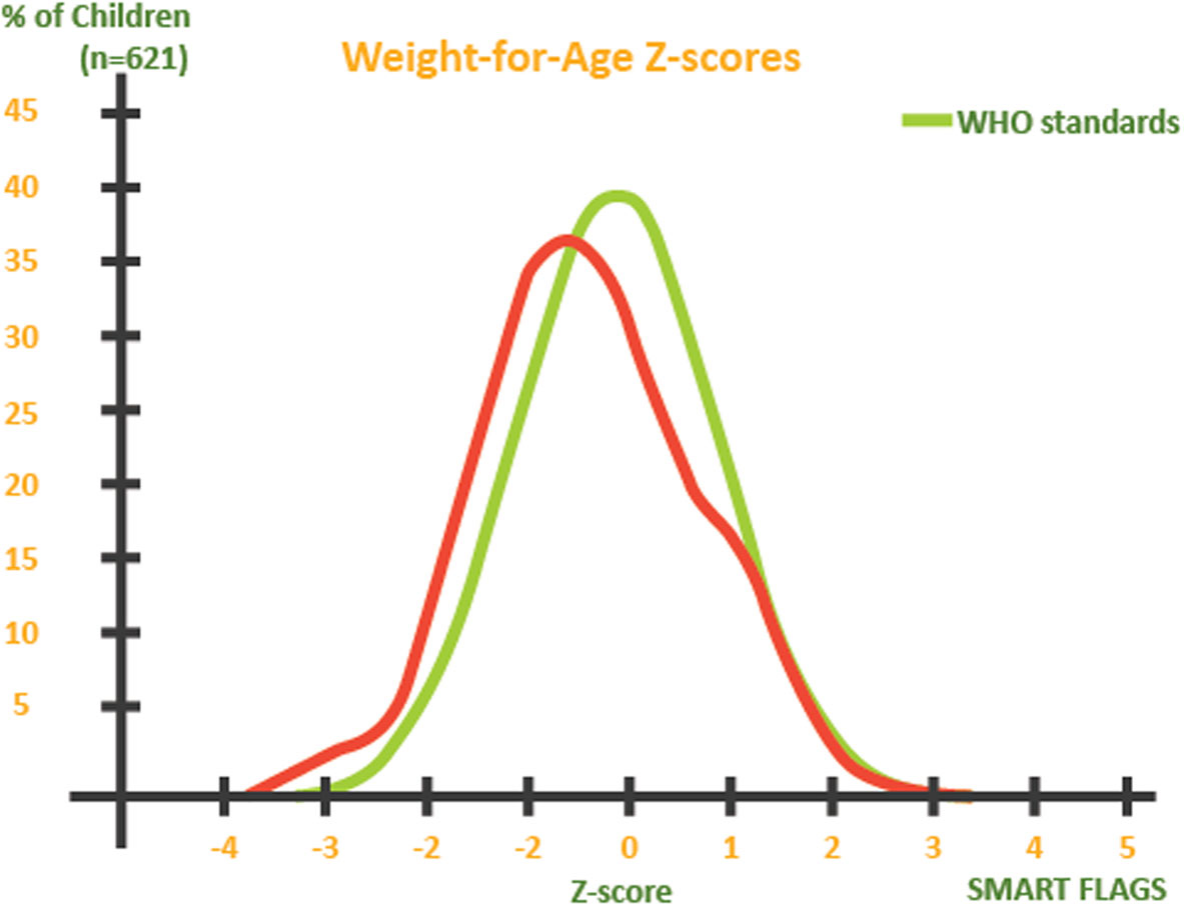
Distribution of weight-for-age z-scores compared to 2006 WHO standards among children below 5 years of age in Yambio County, South Sudan, in 2018 (*n* = 621)

The overall prevalence of stunting based on height-for-age z-scores (<− 2 z-score) was 23.8% (19.1–29.2, 95% CI) while severe stunting (<− 3 z-score) was 7.8% (5.5–10.8, 95% CI). Prevalence of moderate stunting (<− 2 z-score and > = − 3 z-score) was 16.0% (13.0–19.6, 95% C.I.) The distribution of height-for-age z-scores compared to 2006 WHO standards among children below 5 years of age was as shown in Fig. 3. The prevalence of undernutrition based on wasting, underweight and stunting by age and sex were summarized in Table 2.

**Table 2:** Prevalence of undernutrition among under-five children in Yambio County, South Sudan, in 2018

Undernutrition Indices	Sex		Age (Months)		Total
	Male	Female	6 to 23	24 to 59	Frequency (%)
	Frequency (%)	Frequency (%)	Frequency (%)	Frequency (%)	
**Wasting (*n*** **= 615)**					
Overall	11 (1.8)	3 (0.5)	8 (1.3)	6 (1.0)	14 (2.3)
Moderate (−3SD ≤ WHZ < −2SD)	11 (1.8)	3 (0.5)	8 (1.3)	6 (1.0)	14 (2.3)
**Underweight (*n*** **= 621)**					
Overall	16 (2.6)	14 (2.3)	15 (2.4)	15 (2.4)	30 (4.8)
Moderate (−3SD ≤ WAZ < −2SD)	12 (1.9)	11 (1.8)	12 (1.9)	11 (1.8)	23 (3.7)
**Stunting (*n*** **= 597)**					
Overall	82 (13.7)	60 (10.1)	59 (9.9)	83 (13.9)	142 (23.8)
Moderate (−3SD ≤ HAZ < −2SD)	49 (8.2)	46 (7.7)	39 (6.5)	56 (9.4)	95 (16.0)

*HZ* weight-for-height z-scores; *WAZ* weight-for-age z-scores; *HAZ* height-for-age z-scores

### Predictors of undernutrition

Predictors of the three types of undernutrition (stunting, underweight and wasting) were analyzed separately. According to the bivariate analysis results, under-five children of male gender were nearly four times more likely to be wasted than female children (cOR [95% CI], *p*-value: 3.7 [1.02–13.3], *p* = 0.047). The risk of wasting in children aged 6–23 months was 2.3 times higher than those aged 24–59 months, but this increased risk was not statistically significant (cOR [95% CI], *p*-value: 2.3 [0.78–6.6], *p* = 0.136). Children from households of non-residents were more than four times at increased risk of wasting (cOR [95% CI], *p*-value: 4.2 [1.4–12.2] *p* = 0.009).

The risk of underweight increased by 10% for every increase in the household size by one member (cOR [95% CI], p-value: 1.09 [1.01–1.18] *p* = 0.029). The risk of underweight in children aged 6–23 months was similar to those aged 24–59 months (cOR [95% CI], *p*-value: 1.7 [0.83–3.59] *p* = 0.147).

There was no statistically significant differences on risk of stunting among children aged 6–23 months compared to those aged 24–59 months (cOR [95% CI], *p*-value: 1.2 [0.83–1.78] *p* = 0.322. Under-five children from families whose main livelihood activities were agricultural were 2.3 times more likely to be stunted than children from families whose main livelihood activities were non-agricultural (cOR [95% CI], *p*-value: 2.3 [1.28–4.21] *p* = 0.006).

According to the multivariable analysis results, male children were as likely to have wasting as female children (aOR [95% CI], *p*-value: 4.3[0.90–21.01], *p* = 0.068). The risk of wasting in children aged 6–23 months was similar to those aged 24–59 months (aOR [95% CI], *p*-value: 1.4[0.38–5.22, *p* = 0.603). All other predictor variables included in the model were statistically significant (*p* > 0.05). Similarly, none of the predictor variables included in the regression model significantly contributed to underweight among the under-five children in Yambio County (*p* > 0.05).

The risk of stunting in children aged 6–59 months did not differ significantly by age and gender (*p* > 0.05). The risk of stunting among under-five children from families whose main livelihood activities were agricultural was 3.1 times higher than that of children from families whose main livelihood activities were non-agricultural (aOR [95% CI], *p*-value: 3.1 [1.51–6.45] *p* = 0.002). Other independent variables included in the model were not statistically significant (*p* > 0.05). A summary of bivariate and multivariable analysis of the predictors of undernutrition among children below 5 years of age in Yambio County, South Sudan were presented in Table 3.

**Table 3:** Bivariate and multivariable analysis of undernutrition among children below 5 years of age in Yambio County, South Sudan, in 2018 (*N* = 630)

Variables	Wasting				Underweight				Stunting			
	cOR (95% CI)	***P*** value	aOR (95% CI)	***P*** value	cOR (95% CI)	***P*** value	aOR (95% CI)	***P*** value	cOR (95% CI)	***P*** value	aOR (95% CI)	***P*** value
Sex												
Female	Reference											
Male	3.68 (1.02–13.32)	0.047*	4.3 (0.90–21.01)	0.068	1.12 (0.54–2.33)	0.770	1.0 (0.36–2.88)	0.979	1.15 (0.99–2.12)	0.057	1.3 (0.87–2.06)	0.180
Age (Months)												
6–23	2.3 (0.77–6.60)	0.136	1.4 (0.38–5.23)	0.603	1.7 (0.83–3.59)	0.147	1.3 (0.43–4.04)	0.632	1.2 (0.83–1.78)	0.322	1.4 (0.90–2.25)	0.130
24–59	Reference		Reference		Reference		Reference		2.6 (0.96–7.03)	0.060	Reference	
Household head												
Male												
Female	0.68 (0.15–3.06)	0.610	0.57 (0.10–3.23)	0.525	0.44 (0.13–1.46)	0.179	0.76 (0.16–3.58)	0.726	1.12 (0.71–1.77)	0.637	1.2 (0.69–2.03)	0.537
Household size	1.05 (0.94–1.18)	0.385	1.04 (0.91–1.21)	0.525	1.09 (1.01–1.18)	0.029*	1.1 (0.94–1.22)	0.322	1.0 (0.97–1.06)	0.564		
Resident status												
Resident												
Non-resident	4.2 (1.44–12.15)	0.009*	2.9 (0.70–11.85)	0.141	0.79 (0.3–2.11)	0.644	1.1 (0.22–5.42)	0.915	0.89 (0.56–1.43)	0.643		
Main livelihood activity												
Agricultural	Reference		Reference		1.97 (0.59–6.60)	0.274	1.9 (0.39–9.51)	0.424	2.32 (1.28–4.21)	0.006*	3.1 (1.51–6.45)	0.002*
Non-agricultural	1.06 (0.77–1.47)	0.714	1.2 (0.82–1.282)	0.331					Reference		Reference	
Vitamin A Supplementation												
No												
Yes	1.46 (0.42–5.29)	0.566	4.2 (0.36–47.58)	0.251	1.6 (0.64–3.99)	0.311	2.2 (0.39–12.81)	0.364	0.93 (0.62–1.41)	0.739	0.7 (0.38–1.32)	0.275
Measles vaccination												
No												
Yes	0.70 (0.15–3.21)	0.642	0.6 (0.05–6.84)	0.673	1.64 (0.38–7.08)	0.507	1.2 (0.09–16.04)	0.884	1.65 (0.84–3.25)	0.148	2.2 (0.91–5.49)	0.078
Illness												
No												
Yes	1.59 (0.53–4.81)	0.410	1.7 (0.44–6.83)	0.435	2.02 (0.91–4.52)	0.086	1.5 (0.48–4.72)	0.491	1.13 (0.77–1.66)	0.521		
Using Long Lasting Insecticide Treated Nets												
No												
Yes	0.43 (0.15–1.25)	0.122	0.5 (0.09–2.26)	0.339	2.2 (0.82–5.85)	0.115	4.6 (0.85–24.60)	0.077	1.12 (0.74–1.69)	0.601	1.2 (0.68–1.95)	0.598
Deworming												
No												
Yes	0.66 (0.19–2.28)	0.511	0.7 (0.17–2.71)	0.582	1.4 (0.60.32)	0.433	0.8 (0.22–2.52)	0.644	1.08 (0.73–1.61)	0.704	1.4 (0.79–2.35)	0.266

*cOR* Crude Odds ratio; *aOR* Adjusted Odds ratio; *CI* confidence interval; * = statistically significant *p*-value < 0.05

## Discussion

There has been fears that the years of conflict in Yambio County leading to large displacements may have affected families’ ability to raise and harvest crops [23]. We assessed the prevalence and predictors of wasting, underweight and stunting in this conflict setting among 630 children aged from 6 to 59 months. Our study population was drawn from Yambio County, a region that was at the heart of armed clashes and widespread displacement in 2016 and hosts a large population of internally displaced persons. The covariates assessed were child sex, child age, household size, resident status, main livelihood activity, Vitamin A supplementation, immunization status, presence of illness, using long-lasting insecticide treated nets and deworming. The main finding in our study was overall low levels of child undernutrition. We also identified important predictors that were independently associated with the risk of the different aspects of child undernutrition (wasting, underweight and stunting) and quantified the magnitude of the risk that was associated with each characteristic.

According to prevalence thresholds outlined by WHO, our findings on the prevalence of wasting as measured through global acute malnutrition and severe acute malnutrition was very low (< 2.5%) [27]. Acute malnutrition among children aged 6–59 months is a key indicator used routinely to describe the presence and magnitude of humanitarian emergencies. Many nutritional surveys have reported significantly higher prevalence of malnutrition in conflict-affected countries [3, 4]. In 2018, 63% (7.1 million) people in South Sudan were facing acute food insecurity. More than 15% of people in seven out of the ten country’s ten states were malnourished. Across the country almost 3 million children were severely food insecure and more than 1 million were acutely malnourished and almost 280,000 were severely malnourished [28].

Yambio county lies in a livelihood zone of equatorial maize and cassava and is the only region in South Sudan with a bimodal rainfall pattern and two reliable seasons. Compared to the rest of South Sudan, the zone has high potential for crop production due to good soils and higher rainfall amounts. The nutrition profile of Yambio county is likely similar to that of neighbouring Maridi County which lie in the same livelihood zone [29]. A SMART survey in Maridi County in September 2018 reported a global acute malnutrition point prevalence of 5.5%, which is indicative of the low prevalence of wasting in this livelihood zone [30]. Our study was done in October and November, which is the harvesting period of cereals and the all year round cassava [29]. Considering that acute malnutrition in South Sudan has been shown to vary with the harvesting season, it is conceivable that the malnutrition prevalence could be higher during the pre-harvest season in Yambio County [13].

Wasting provides information on the proportion of children in a certain age range in a humanitarian setting who are classified with low weight-for-height and/or oedema. Wasting is a good indicator for short term (recent) nutritional history in under-five children as they are particularly vulnerable. Our findings indicate that the levels are of very low public health concern at this moment and immediate actions beyond continued surveillance may not be necessary in Yambio county. We found that male children were not at increased risk of wasting. Several other studies in similar and different settings have reported increased vulnerability of male children compared to female children aged 6–59 months [9, 14, 31].

We found that the level of underweight prevalence was low (< 5%) according to the WHO standards [27]. The magnitude of the effect of household size on underweight increased in a dose-response way, with increasing household size negatively impacting underweight among under-five children though this was not statistically significant. Findings of household size being negatively associated with underweight was reported in a study in Ethiopia [32]. Household size affects household food security which has a direct link with nutritional status. We did not find age as a significant predictor of underweight. This was contrast findings to a study in Kenya which reported a higher risk of underweight at 2 years of age [15].

Using the WHO standards, prevalence of stunting in our study was categorized as high (20 to < 30%). Globally, stunting is the most prevalent form of undernutrition with estimates of 21.9% or 149 million under-five children in 2018 [33]. Our findings on the prevalence of stunting were therefore congruent with the global rate [1, 33]. Many studies in the neighboring countries have reported higher stunting prevalence levels. Stunting was estimated at 26% in Kenya based on a national survey in 2014 [34], at 24.9% in West Gojjam Zone, Northwest Ethiopia [35], at 44.7% in east Gojjam zone, Northwest Ethiopia [8], at 31% in Somalia [36], at 42.5% in North Sudan [37] and 42.7% in Democratic Republic of Congo [38].

We found that stunting did not differ significantly by age. Significant findings of stunting of children in their second year of life were reported in a study in Kenya [15]. This may be associated with timing of starting the complementary feeding and type of foods introduced as reported in a study in Ethiopia [8]. A study in Ethiopia among school-age children reported association of stunting with child age [39]. Stunting in our study was associated with children from families whose main livelihood was agricultural. Evidence shows that agriculture, including subsistence farming, can be leveraged to enhance nutritional status, especially production of nutrition-rich crops with diversification [40, 41]. Empowerment in nutrition knowledge, especially in women, is crucial in improving nutritional outcomes in communities whose main livelihood is agriculture [41]. The main crops grown in both seasons at Yambio County were maize, sorghum, cassava, groundnut and sweet potatoes [29]. Diversification of the agricultural production towards fruits and vegetables can potentially improve nutrient intake and nutritional outcomes.

## Conclusion

In our study, wasting prevalence was very low, underweight prevalence was classified as low while stunting prevalence was categorized as high. The county lies in the only livelihood region in South Sudan with bimodal reliable rainfall pattern and it seems that the impact of the 2016 conflicts that lead to large displacements may not have greatly affected the under-five undernutrition. After adjusting for other factors, male child sex and older child’s age were associated with wasting while younger child’s age and agricultural livelihood were associated factors with stunting. At bivariate level, household size and younger child’s age were associated with underweight, but none of the evaluated variables was associated with underweight after controlling for other risk factors. Interventions targeted at improving food diversity, increasing knowledge on nutrition and enhancing resilience in male children might reduce undernutrition. Investment in continued surveillance of nutritional status among under-five children should be the main focus in the short-term.

## Data Availability

All data generated and analyzed during this study is included in this manuscript and has been attached as a supplementary file. The dataset is also available from the corresponding author on request.

## Abbreviations

ENA: Emergency Nutrition Assessment
HAZ: Height-for-Age Z-score
SMART: Standardized Monitoring and Assessment of Relief and Transition
WAZ: Weight-for-Age Z-score
WHO: World Health Organization
WHZ: Weight-for-Height Z-score

## References

[1] Development Initiatives, “2018 Global Nutrition Report,” Development Initiatives, Bristol, UK, 2018. [Online]. Available: https://globalnutritionreport.org/reports/global-nutrition-report-2018/burden-malnutrition/.

[2] Black RE, Allen LH, Bhutta ZA, Caulfield LE, de Onis M, Ezzati M, Mathers C, Rivera J (2008). Maternal and child undernutrition: global and regional exposures and health consequences. Lancet.

[3] FAO/WFP, “Monitoring food security in countries with conflict situations,” The Food and Agriculture Organization of the United Nations and The World Food Programme, Rome, Italy, 3, Jan. 2018.

[4] FSIN, “Global report on food crises 2017,” Food security information network, Rome, Italy, Mar. 2017. Accessed: 17 Apr 2020. [Online]. Available: http://www.fao.org/emergencies/resources/documents/resources-detail/en/c/876564/.

[5] FSIN, “Global report on food crises 2018,” Food security information network, Mar. 2018. Accessed: 17 Apr 2020. [Online]. Available: http://www.fao.org/emergencies/resources/documents/resources-detail/en/c/1107313/.

[6] Gakidou E, Afshin A, Abajobir AA, Abate KH, Abbafati C, Abbas KM, Abd-Allah F, Abdulle AM, Abera SF, Aboyans V, Abu-Raddad LJ, Abu-Rmeileh NME, Abyu GY, Adedeji IA, Adetokunboh O, Afarideh M, Agrawal A, Agrawal S, Ahmadieh H, Ahmed MB, Aichour MTE, Aichour AN, Aichour I, Akinyemi RO, Akseer N, Alahdab F, al-Aly Z, Alam K, Alam N, Alam T, Alasfoor D, Alene KA, Ali K, Alizadeh-Navaei R, Alkerwi A', Alla F, Allebeck P, al-Raddadi R, Alsharif U, Altirkawi KA, Alvis-Guzman N, Amare AT, Amini E, Ammar W, Amoako YA, Ansari H, Antó JM, Antonio CAT, Anwari P, Arian N, Ärnlöv J, Artaman A, Aryal KK, Asayesh H, Asgedom SW, Atey TM, Avila-Burgos L, Avokpaho EFGA, Awasthi A, Azzopardi P, Bacha U, Badawi A, Balakrishnan K, Ballew SH, Barac A, Barber RM, Barker-Collo SL, Bärnighausen T, Barquera S, Barregard L, Barrero LH, Batis C, Battle KE, Baumgarner BR, Baune BT, Beardsley J, Bedi N, Beghi E, Bell ML, Bennett DA, Bennett JR, Bensenor IM, Berhane A, Berhe DF, Bernabé E, Betsu BD, Beuran M, Beyene AS, Bhansali A, Bhutta ZA, Bicer BK, Bikbov B, Birungi C, Biryukov S, Blosser CD, Boneya DJ, Bou-Orm IR, Brauer M, Breitborde NJK, Brenner H, Brugha TS, Bulto LNB, Butt ZA, Cahuana-Hurtado L, Cárdenas R, Carrero JJ, Castañeda-Orjuela CA, Catalá-López F, Cercy K, Chang HY, Charlson FJ, Chimed-Ochir O, Chisumpa VH, Chitheer AA, Christensen H, Christopher DJ, Cirillo M, Cohen AJ, Comfort H, Cooper C, Coresh J, Cornaby L, Cortesi PA, Criqui MH, Crump JA, Dandona L, Dandona R, das Neves J, Davey G, Davitoiu DV, Davletov K, de Courten B, Defo BK, Degenhardt L, Deiparine S, Dellavalle RP, Deribe K, Deshpande A, Dharmaratne SD, Ding EL, Djalalinia S, Do HP, Dokova K, Doku DT, Donkelaar A, Dorsey ER, Driscoll TR, Dubey M, Duncan BB, Duncan S, Ebrahimi H, el-Khatib ZZ, Enayati A, Endries AY, Ermakov SP, Erskine HE, Eshrati B, Eskandarieh S, Esteghamati A, Estep K, Faraon EJA, Farinha CSS, Faro A, Farzadfar F, Fay K, Feigin VL, Fereshtehnejad SM, Fernandes JC, Ferrari AJ, Feyissa TR, Filip I, Fischer F, Fitzmaurice C, Flaxman AD, Foigt N, Foreman KJ, Frostad JJ, Fullman N, Fürst T, Furtado JM, Ganji M, Garcia-Basteiro AL, Gebrehiwot TT, Geleijnse JM, Geleto A, Gemechu BL, Gesesew HA, Gething PW, Ghajar A, Gibney KB, Gill PS, Gillum RF, Giref AZ, Gishu MD, Giussani G, Godwin WW, Gona PN, Goodridge A, Gopalani SV, Goryakin Y, Goulart AC, Graetz N, Gugnani HC, Guo J, Gupta R, Gupta T, Gupta V, Gutiérrez RA, Hachinski V, Hafezi-Nejad N, Hailu GB, Hamadeh RR, Hamidi S, Hammami M, Handal AJ, Hankey GJ, Hanson SW, Harb HL, Hareri HA, Hassanvand MS, Havmoeller R, Hawley C, Hay SI, Hedayati MT, Hendrie D, Heredia-Pi IB, Hernandez JCM, Hoek HW, Horita N, Hosgood HD, Hostiuc S, Hoy DG, Hsairi M, Hu G, Huang JJ, Huang H, Ibrahim NM, Iburg KM, Ikeda C, Inoue M, Irvine CMS, Jackson MD, Jacobsen KH, Jahanmehr N, Jakovljevic MB, Jauregui A, Javanbakht M, Jeemon P, Johansson LRK, Johnson CO, Jonas JB, Jürisson M, Kabir Z, Kadel R, Kahsay A, Kamal R, Karch A, Karema CK, Kasaeian A, Kassebaum NJ, Kastor A, Katikireddi SV, Kawakami N, Keiyoro PN, Kelbore SG, Kemmer L, Kengne AP, Kesavachandran CN, Khader YS, Khalil IA, Khan EA, Khang YH, Khosravi A, Khubchandani J, Kiadaliri AA, Kieling C, Kim JY, Kim YJ, Kim D, Kimokoti RW, Kinfu Y, Kisa A, Kissimova-Skarbek KA, Kivimaki M, Knibbs LD, Knudsen AK, Kopec JA, Kosen S, Koul PA, Koyanagi A, Kravchenko M, Krohn KJ, Kromhout H, Kumar GA, Kutz M, Kyu HH, Lal DK, Lalloo R, Lallukka T, Lan Q, Lansingh VC, Larsson A, Lee PH, Lee A, Leigh J, Leung J, Levi M, Levy TS, Li Y, Li Y, Liang X, Liben ML, Linn S, Liu P, Lodha R, Logroscino G, Looker KJ, Lopez AD, Lorkowski S, Lotufo PA, Lozano R, Lunevicius R, Macarayan ERK, Magdy Abd el Razek H, Magdy Abd el Razek M, Majdan M, Majdzadeh R, Majeed A, Malekzadeh R, Malhotra R, Malta DC, Mamun AA, Manguerra H, Mantovani LG, Mapoma CC, Martin RV, Martinez-Raga J, Martins-Melo FR, Mathur MR, Matsushita K, Matzopoulos R, Mazidi M, McAlinden C, McGrath JJ, Mehata S, Mehndiratta MM, Meier T, Melaku YA, Memiah P, Memish ZA, Mendoza W, Mengesha MM, Mensah GA, Mensink GBM, Mereta ST, Meretoja TJ, Meretoja A, Mezgebe HB, Micha R, Millear A, Miller TR, Minnig S, Mirarefin M, Mirrakhimov EM, Misganaw A, Mishra SR, Mohammad KA, Mohammed KE, Mohammed S, Mohan MBV, Mokdad AH, Monasta L, Montico M, Moradi-Lakeh M, Moraga P, Morawska L, Morrison SD, Mountjoy-Venning C, Mueller UO, Mullany EC, Muller K, Murthy GVS, Musa KI, Naghavi M, Naheed A, Nangia V, Natarajan G, Negoi RI, Negoi I, Nguyen CT, Nguyen QL, Nguyen TH, Nguyen G, Nguyen M, Nichols E, Ningrum DNA, Nomura M, Nong VM, Norheim OF, Norrving B, Noubiap JJN, Obermeyer CM, Ogbo FA, Oh IH, Oladimeji O, Olagunju AT, Olagunju TO, Olivares PR, Olsen HE, Olusanya BO, Olusanya JO, Opio JN, Oren E, Ortiz A, Ota E, Owolabi MO, PA M, Pacella RE, Pana A, Panda BK, Panda-Jonas S, Pandian JD, Papachristou C, Park EK, Parry CD, Patten SB, Patton GC, Pereira DM, Perico N, Pesudovs K, Petzold M, Phillips MR, Pillay JD, Piradov MA, Pishgar F, Plass D, Pletcher MA, Polinder S, Popova S, Poulton RG, Pourmalek F, Prasad N, Purcell C, Qorbani M, Radfar A, Rafay A, Rahimi-Movaghar A, Rahimi-Movaghar V, Rahman MHU, Rahman MA, Rahman M, Rai RK, Rajsic S, Ram U, Rawaf S, Rehm CD, Rehm J, Reiner RC, Reitsma MB, Remuzzi G, Renzaho AMN, Resnikoff S, Reynales-Shigematsu LM, Rezaei S, Ribeiro AL, Rivera JA, Roba KT, Rojas-Rueda D, Roman Y, Room R, Roshandel G, Roth GA, Rothenbacher D, Rubagotti E, Rushton L, Sadat N, Safdarian M, Safi S, Safiri S, Sahathevan R, Salama J, Salomon JA, Samy AM, Sanabria JR, Sanchez-Niño MD, Sánchez-Pimienta TG, Santomauro D, Santos IS, Santric Milicevic MM, Sartorius B, Satpathy M, Sawhney M, Saxena S, Schmidt MI, Schneider IJC, Schutte AE, Schwebel DC, Schwendicke F, Seedat S, Sepanlou SG, Serdar B, Servan-Mori EE, Shaddick G, Shaheen A, Shahraz S, Shaikh MA, Shamsipour M, Shamsizadeh M, Shariful Islam SM, Sharma J, Sharma R, She J, Shen J, Shi P, Shibuya K, Shields C, Shiferaw MS, Shigematsu M, Shin MJ, Shiri R, Shirkoohi R, Shishani K, Shoman H, Shrime MG, Sigfusdottir ID, Silva DAS, Silva JP, Silveira DGA, Singh JA, Singh V, Sinha DN, Skiadaresi E, Slepak EL, Smith DL, Smith M, Sobaih BHA, Sobngwi E, Soneji S, Sorensen RJD, Sposato LA, Sreeramareddy CT, Srinivasan V, Steel N, Stein DJ, Steiner C, Steinke S, Stokes MA, Strub B, Subart M, Sufiyan MB, Suliankatchi RA, Sur PJ, Swaminathan S, Sykes BL, Szoeke CEI, Tabarés-Seisdedos R, Tadakamadla SK, Takahashi K, Takala JS, Tandon N, Tanner M, Tarekegn YL, Tavakkoli M, Tegegne TK, Tehrani-Banihashemi A, Terkawi AS, Tesssema B, Thakur JS, Thamsuwan O, Thankappan KR, Theis AM, Thomas ML, Thomson AJ, Thrift AG, Tillmann T, Tobe-Gai R, Tobollik M, Tollanes MC, Tonelli M, Topor-Madry R, Torre A, Tortajada M, Touvier M, Tran BX, Truelsen T, Tuem KB, Tuzcu EM, Tyrovolas S, Ukwaja KN, Uneke CJ, Updike R, Uthman OA, van Boven JFM, Varughese S, Vasankari T, Veerman LJ, Venkateswaran V, Venketasubramanian N, Violante FS, Vladimirov SK, Vlassov VV, Vollset SE, Vos T, Wadilo F, Wakayo T, Wallin MT, Wang YP, Weichenthal S, Weiderpass E, Weintraub RG, Weiss DJ, Werdecker A, Westerman R, Whiteford HA, Wiysonge CS, Woldeyes BG, Wolfe CDA, Woodbrook R, Workicho A, Xavier D, Xu G, Yadgir S, Yakob B, Yan LL, Yaseri M, Yimam HH, Yip P, Yonemoto N, Yoon SJ, Yotebieng M, Younis MZ, Zaidi Z, Zaki MES, Zavala-Arciniega L, Zhang X, Zimsen SRM, Zipkin B, Zodpey S, Lim SS, Murray CJL (2017). Global, regional, and national comparative risk assessment of 84 behavioural, environmental and occupational, and metabolic risks or clusters of risks, 1990–2016: a systematic analysis for the global burden of disease study 2016. Lancet.

[7] E. N. Odjidja and S. Hakizimana, “Data on acute malnutrition and mortality among under-5 children of pastoralists in a humanitarian setting: a cross-sectional Standardized Monitoring and Assessment of Relief and Transitions Study,” *BMC Res Notes*, vol. 12, 2019, doi: 10.1186/s13104-019-4475-x. 10.1186/s13104-019-4475-x PMC6642480 31324270

[8] A. Zeray, G. D. Kibret, and C. T. Leshargie, “Prevalence and associated factors of undernutrition among under-five children from model and non-model households in east Gojjam zone, Northwest Ethiopia: a comparative cross-sectional study,” *BMC Nutr*, vol. 5, no. 1, p. 27, 2019, doi: 10.1186/s40795-019-0290-y. 10.1186/s40795-019-0290-y PMC7050904 32153940

[9] A. Díez Navarro, S. Marrodán, M. Dolores, and G. de A. Arriba, “Female eco-stability and severe malnutrition in children: Evidence from humanitarian aid interventions of Action Against Hunger in African, Asian and Latin American countries,” *Nutricion Clinica y Dietetica Hospitalaria*, no. 4, pp. 127–134, 2017, doi: 10.12873/374dnavarro.

[10] Meshram II, Arlappa N, Balakrishna N, Rao KM, Laxmaiah A, Brahmam GNV (2012). Trends in the prevalence of undernutrition, nutrient and food intake and predictors of undernutrition among under five year tribal children in India. Asia Pacific J Clinical Nutrition.

[11] Hovhannisyan L, Demirchyan A, Petrosyan V (2014). Estimated prevalence and predictors of undernutrition among children aged 5–17 months in Yerevan, Armenia. Public Health Nutr.

[12] Egata G, Berhane Y, Worku A (2014). Predictors of acute undernutrition among children aged 6 to 36 months in east rural Ethiopia: a community based nested case - control study. BMC Pediatr.

[13] Hogan C, Golden K, Kopplow R, Ferguson E (2019). Analysis of trends in SMART nutrition survey data from South Sudan between 2004 and 2016. South Sudan Medical J.

[14] Medhin G (2010). Prevalence and predictors of undernutrition among infants aged six and twelve months in Butajira, Ethiopia: The P-MaMiE Birth Cohort. BMC Public Health.

[15] Bloss E, Wainaina F, Bailey RC (2004). Prevalence and predictors of underweight, stunting, and wasting among children aged 5 and under in Western Kenya. J Trop Pediatr.

[16] Darsene H, Geleto A, Gebeyehu A, Meseret S (2017). Magnitude and predictors of undernutrition among children aged six to fifty nine months in Ethiopia: a cross sectional study. Arch Public Health.

[17] Pelletier DL, Frongillo EA, Habicht JP (1993). Epidemiologic evidence for a potentiating effect of malnutrition on child mortality. Am J Public Health.

[18] Morgan G (1997). What, if any, is the effect of malnutrition on immunological competence?. Lancet.

[19] United Nations Children’s Fund (UNICEF), “Malnutrition,” 2020. https://data.unicef.org/topic/nutrition/malnutrition/.

[20] McFie J, Welbourn HF (1962). Effect of malnutrition in infancy on the development of bone, muscle and fat. J Nutr.

[21] Action Against Hunger, “SMART,” Action Against Hunger, 2018. https://actionagainsthunger.ca/program-areas/smart/ (accessed 18 Apr 2020).

[22] Central Bureau of Statistics, “Sudan - Population and Housing Census 2008,” Central Bureau of Statistics - Sudan Government of National Unity Southern Sudan Commission for Statistics and Evaluation, Sudan, Census report, 2009. Accessed: 16 Dec 2019. [Online]. Available: http://www.ilo.org/surveydata/index.php/catalog/1360.

[23] USAID REACH Initiative, “South Sudan Displacement Crisis: Yambio Town Road Monitoring - Yambio County, Western Equatoria State, South Sudan.,” 2018. https://m.reliefweb.int/report/2704244/south-sudan/south-sudan-displacement-crisis-yambio-town-road-monitoring-yambio-county-3.

[24] National Bureau of Statistics (2012). “South Sudan Statistical Yearbook 2011,” Juba, Soth Sudan.

[25] World Health Organization (2006). WHO child growth standards : length/height-for-age, weight-for-age, weight-for-length, weight -for-height and body mass index-for-age : methods and development. World Health Organization.

[26] StataCorp (2019). Stata Statistical Software: Release 16.

[27] de Onis M, Borghi E, Arimond M, Webb P, Croft T, Saha K, de-Regil LM, Thuita F, Heidkamp R, Krasevec J, Hayashi C, Flores-Ayala R (2019). Prevalence thresholds for wasting, overweight and stunting in children under 5 years. Public Health Nutr.

[28] United Nations Children’s Fund (UNICEF), “Childhood under attack. The staggering impact of South Sudan’s crisis on children.,” 2017. [Online]. Available: https://www.unicef.org/media/files/UNICEF_South_Sudan_Report_Childhood_under_Attack_15Dec_FINAL.pdf.

[29] FEWS NET, “South Sudan Livelihood Zones and Descriptions (2013),” Famine Early Warning Systems Network (FEWS NET), Washington, Aug. 2013. Accessed: 18 Apr 2020. [Online]. Available: http://efd.org/reports/south-sudan-livelihood-zones-and-descriptions-2013/.

[30] UNICEF, “Preliminary Report of SMART Nutrition Survey in the former Maridi County, former Western Equitoria State from 24th August to 3rd September 2018.,” The United Nations Children’s Fund, South Sudan, 2018. Accessed: 18 Apr 2020. [Online]. Available: https://www.humanitarianresponse.info/en/operations/south-sudan/document/2018-smart-surveys-reports.

[31] McDonald CM, Kupka R, Manji KP, Okuma J, Bosch RJ, Aboud S, Kisenge R, Spiegelman D, Fawzi WW, Duggan CP (2012). Predictors of stunting, wasting and underweight among Tanzanian children born to HIV-infected women. Eur J Clin Nutr.

[32] Assefa H, Belachew T, Negash L (2013). Socioeconomic Factors Associated with Underweight andStunting among Adolescents of Jimma Zone, South WestEthiopia: A Cross-Sectional Study. Hindawi.

[33] United Nations Children’s Fund, World Health Organization, and World Bank Group, “UNICEF/WHO/The World Bank Group Joint child malnutrition estimates - levels and trends in child malnutrition. Key findings of the 2019 edition,” World Health Organization, 2019. Accessed: 20 Apr 2020. [Online]. Available: http://www.who.int/nutrition/publications/jointchildmalnutrition-2019-estimates/en/.

[34] Kenya National Bureau of Statistics, “Kenya - Demographic and Health Survey 2014 (DHS 2014 / KDHS 2014),” Kenya National Bureau of Statistics, Government of Kenya, Nairobi, Kenya, 2014. Accessed: 24 Dec 2019. [Online].

[35] Amare D, Negesse A, Tsegaye B, Assefa B, Ayenie B. Prevalence of undernutrition and its associated factors among children below five years of age in Bure town, west Gojjam zone, Amhara National Regional State, Northwest Ethiopia. Adv Public Health. 2016;2016.

[36] Kinyoki DK, Berkley JA, Moloney GM, Kandala N-B, Noor AM (2015). Predictors of the risk of malnutrition among children under the age of 5 years in Somalia. Public Health Nutr.

[37] Sulaiman AA (2018). Prevalence and determinants of undernutrition among children under 5-year-old in rural areas: A cross-sectional survey in North Sudan. J Fam Med Prim Care.

[38] Akombi BJ, Agho KE, Merom D, Renzaho AM, Hall JJ (2017). Child malnutrition in sub-Saharan Africa: A meta-analysis of demographic and health surveys (2006–2016). PloS one.

[39] Lisanu Mazengia A, Andargie Biks G. Predictors of stunting among school-age children in northwestern Ethiopia. J Nutr Metab. 2018;2018. 10.1155/2018/7521751 PMC6171210 30327729

[40] Otterbach S, Rogan M (2017). Spatial differences in stunting and household agricultural production in South Africa:(re-) examining the links using national panel survey data.

[41] Pandey VL, Dev SM, Jayachandran U (2016). Impact of agricultural interventions on the nutritional status in South Asia: a review. Food Policy.

